# Stakeholder engagement in eight comparative effectiveness trials in African Americans and Latinos with asthma

**DOI:** 10.1186/s40900-022-00399-x

**Published:** 2022-11-24

**Authors:** Tiffany Dy, Winifred J. Hamilton, C. Bradley Kramer, Andrea Apter, Jerry A. Krishnan, James W. Stout, Stephen J. Teach, Alex Federman, John Elder, Tyra Bryant-Stephens, Rebecca J. Bruhl, Shawni Jackson, Kaharu Sumino

**Affiliations:** 1 Division of Allergy and Immunology, Department of Internal Medicine, Washington University School of Medicine in St. Louis, 660 S. Euclid Ave, CB 8122, St. Louis, MO 63110 USA; 2 Environmental Health Service, Department of Family and Community Medicine, Baylor College of Medicine, Houston, TX USA; 3 Department of Pediatrics and Health Services, University of Washington, Seattle, WA USA; 4 Division of Pulmonary, Allergy, and Critical Care Medicine, Department of Medicine, Perelman School of Medicine, University of Pennsylvania, Philadelphia, PA USA; 5 Division of Pulmonary, Critical Care, Sleep, and Allergy, Department of Medicine, University of Illinois Chicago and Population Health Sciences Program, University of Illinois Hospital and Health Sciences System, Chicago, IL USA; 6 Division of Emergency Medicine and the Department of Pediatrics, Children’s National Hospital, Washington, DC USA; 7 Division of General Internal Medicine, Icahn School of Medicine at Mount Sinai, New York, NY USA; 8 Institute for Behavioral and Community Health, School of Public Health, San Diego State University, San Diego, CA USA; 9 Division of General Medical Sciences, Department of Medicine, Washington University School of Medicine in St. Louis, St. Louis, MO USA; 10 Division of Pulmonary and Critical Care, Department of Internal Medicine, Washington University School of Medicine in St. Louis, St. Louis, MO USA; 11 Public Health - Seattle & King County, Seattle, WA USA

**Keywords:** Asthma research, Stakeholder engagement, Comparative effectiveness research, Patient-oriented research

## Abstract

**Background:**

The effects of stakeholder engagement, particularly in comparative effectiveness trials, have not been widely reported. In 2014, eight comparative effectiveness studies targeting African Americans and Hispanics/Latinos with uncontrolled asthma were funded by the Patient-Centered Outcomes Research Institute (PCORI) as part of its Addressing Disparities Program. Awardees were required to meaningfully involve patients and other stakeholders. Using specific examples, we describe how these stakeholders substantially changed the research protocols and in other ways participated meaningfully as full partners in the development and conduct of the eight studies.

**Methods:**

Using the method content analysis of cases, we identified themes regarding the types of stakeholders, methods of engagement, input from the stakeholders, changes made to the research protocols and processes, and perceived benefits and challenges of the engagement process. We used summaries from meetings of the eight teams, results from an engagement survey, and the final research reports as our data source to obtain detailed information. The descriptive data were assessed by multiple reviewers using inductive and deductive qualitative methods and discussed in the context of engagement literature.

**Results:**

Stakeholders participated in the planning, conduct, and dissemination phases of all eight asthma studies. All the studies included clinicians and community representatives as stakeholders. Other stakeholders included patients with asthma, their caregivers, advocacy organizations, and health-system representatives. Engagement was primarily by participation in advisory boards, although six of the eight studies (75%) also utilized focus groups and one-on-one interviews. Difficulty finding a time and location to meet was the most reported challenge to engagement, noted by four of the eight teams (50%). Other reported challenges and barriers to engagement included recruitment of stakeholders, varying levels of enthusiasm among stakeholders, controlling power dynamics, and ensuring that stakeholder involvement was reflected and had true influence on the project.

**Conclusion:**

Engagement-driven modifications led to specific changes in study design and conduct that were felt to have increased enrollment and the general level of trust and support of the targeted communities. The level of interaction described, between investigators and stakeholders in each study and between investigator-stakeholder groups, is—we believe—unprecedented and may provide useful guidance for other studies seeking to improve the effectiveness of community-driven research.

**Supplementary Information:**

The online version contains supplementary material available at 10.1186/s40900-022-00399-x.

## Plain English summary

The goal of comparative clinical effectiveness research is to compare healthcare options and learn which work best for patients depending on their preferences and circumstances. Research efforts can be more effective when researchers engage stakeholders, such as patients, healthcare providers, and other members of the community—especially those communities or groups targeted by the planned research. Stakeholders can give their input throughout the research process to make sure the study will address questions and concerns that are most important and useful for participants. In 2014, the PCORI funded eight research studies that evaluated various ways to help African Americans and Hispanics/Latinos with poorly controlled asthma. These groups are underrepresented in asthma research but have higher rates of and more severe asthma for reasons that are poorly understood. The goal of this report is to show how stakeholders—including patients with asthma from these underrepresented groups, healthcare providers who care for patients with asthma, key representatives from the communities and others—participated as full partners in the eight studies and helped to improve the overall quality of the research and the relationship between the researchers and the community.

**Supplementary Information:**

The online version contains supplementary material available at 10.1186/s40900-022-00399-x.

## Background

In traditional clinical research, patients participate solely as study enrollees and are passive audiences for research results. However, there is a need and growing interest in health and medical research to include patients as active members of research teams, where they participate as advisors, collaborators, and co-investigators [1, 2]. Stakeholders for medical research are defined as individuals from the community with a vested interest in a study, including patients, advocacy groups, caregivers, community groups, healthcare providers, or others who are impacted by the research and the planned intervention [1]. Ideally, these stakeholders are involved from the early stages of study planning through implementation and dissemination of results. Community-based participatory research (CBPR) already has a history of partnering with stakeholders and seeks to involve community members and academic researchers equitably in all phases of the research process [3–6]. As Woolf et al. note:

Stakeholder engagement elevates the moral plane of research by showing respect to patients and vulnerable populations, treating stakeholders as coequal partners, and minimizing the potential for the research process to alienate patients and communities [7].

Clinical trial participation still remains low in African-American and Latino communities and it has been reported that CBPR methodology may particularly be effective in recruitment and retention of underrepresented groups in research [8].

A variety of publications have described possible approaches and best practices for stakeholder engagement. In 1997, the Centers for Disease Control and Prevention, the National Institutes of Health, and the Agency for the Toxic Substances Disease Registry (part of the U.S. Department of Health and Human Services) acknowledged the need to provide guidance on community involvement in research and published the first edition of “Principles of Community Engagement.” Based on these recommendations and with input from community members across the United States, the Association of American Medical Colleges (AAMC) Collaborative for Health Equity endorse *10 Principles of Trustworthiness* (Additional file 1) [9]. These principles point out key attitudes for engagement necessary for a productive relationship. For example, #1 is “The community is already educated; that’s why it doesn’t trust you.” This point homes in on the fact that historically many researchers have approached CBPR through the lens of educating the community about the value of research, largely failing to acknowledge and benefit from the knowledge and expertise of the community. Thus, there is a need to understand how to carry forward these principles for trustworthiness and engagement into practice. Faulkner et al. identified gaps in engagement in medicine development research, such as suboptimal representation of participants, genuine empowerment of stakeholders, transparency of roles, scope of involvement, and communication and feedback [10]. Harmsen et al. observed that despite valuing patient involvement, scientific norms established by researchers’ education and background were a barrier for researchers. Researchers often view scientific evidence as superior and therefore feel a need to “educate” rather than “engage” when trying to meaningfully incorporate patients’ knowledge in the decision-making process [11].

With the establishment of the Patient-Centered Outcomes Research Institute (PCORI) in 2010 as part of the Patient Protection and Affordable Care Act, PCORI began requiring that stakeholders be included as full partners in setting research priorities, forming research questions, and shaping the design, funding, implementation, and dissemination of research studies [7]. PCORI advocates that early and continued involvement of stakeholders throughout a study can lead to improved protocols and greater use and uptake of research results by patients in target populations. In 2014, eight comparative effectiveness research studies based in different urban areas throughout the United States were funded by PCORI’s Addressing Disparities Program under a funding announcement titled “Treatment Options for African Americans and Hispanics/Latinos with Uncontrolled Asthma” (Table 1). All were required to meaningfully involve stakeholders throughout their study. The research groups engaged stakeholders with a vested interest in African American and Hispanic/Latino communities affected by asthma during all study phases of design, conduct, and dissemination as directed by PCORI. The groups also met monthly to collaborate and exchange ideas for optimizing stakeholder engagement.

**Table 1:** Eight research groups funded under a PCORI Asthma Disparities Program for African Americans and Latinos

Project title	Location	Principal investigator	Study population	Study design
*ASIST* (Asthma Symptom-based adjustment of Inhaled Steroid Therapy in African American children)	St. Louis, MO	Kaharu Sumino, MD, MPH	206 African American children 6–17 yo with mild to moderate asthma	Randomized, open-label, parallel group pragmatic trial with randomization to receive either symptom-based adjustment or physician-based adjustment of asthma medications. PMID: 31371165 [33]
*BEAMS* (Breath with Ease: A Unique Approach to Managing Stress)	Washington, DC	Stephen Teach, MD, MPH	217 African American child-parent pairs. Children were 4-17yo with asthma. All had public insurance	Single-blind, prospective, RCT comparing usual care (guideline-based care with education and short-term care coordination) with usual care plus parental stress management. PMID: 31545115 [34]
Using Information Technology to Improve Access, Communication and Asthma in African American and Hispanic/Latino Adults	Philadelphia, PA	Andrea Apter, MD	301 African American and Hispanic/Latino adults with moderate to severe asthma	RCT comparing asthma control in patients receiving patient portal training plus home visits from CHW and patient portal training alone. PMID: 31181221 [35]
*SAMBA* (supporting asthma self-management behaviors in aging adults)	New York, NY	Alex Federman, MD, MPH	405 adults with moderate to severe asthma age 60 and older	Pragmatic RCT examining effectiveness of home and clinic-based program (SAMBA) compared with usual care for patients with moderate to severe asthma. PMID: 31180474 [36]
*CHICAGO* (Coordinated Healthcare Interventions for Childhood Asthma Gaps in Outcomes)	Chicago, IL	Jerry Krishnan, MD, PhD	373 children with asthma age 5–11 yo presenting to the ED with uncontrolled asthma	Multicenter trial comparing 3 discharge approaches for children presenting to ED with uncontrolled asthma (guideline based, ED only discharge instructions; guideline-based ED discharge instructions + home visits; enhanced usual care). PMID: 28366780 [37]
*HIITBAC* (Houston Home-based Integrated Intervention Targeting Better Asthma Control) for African Americans	Houston, TX	Winifred Hamilton, MS, PhD	263 African American adults with poorly controlled asthma	Randomized pragmatic trial comparing effectiveness of enhanced clinical care with and without home visits on asthma outcomes. PMID: 32151753 [13]
*G2P* (Guidelines to Practice: Reducing Asthma Health Disparities through Guideline Implementation)	Seattle, WA	James Stout, MD, MPH	551 children and adults with Medicaid 5–75 years of age with not well or poorly controlled asthma	RCT comparing effectiveness of CHW home visits with no CHW home visits on asthma outcomes. PMID: 27789250 [38]
*Respiro Sano* (Comparing Programs to Improve Asthma Control and Quality of Life for Latino Youth Living in Rural Areas and Their Caregivers)	San Diego, CA	John Elder, PhD, MPH	400 Latino children 6–17 years of age with mild, moderate, or severe persistent asthma in rural areas near California-Mexico border	2 × 2 factorial study nested within community intervention comparing approaches to improving asthma outcomes and quality of life (community program only, community program plus family program, community program plus clinic program, community program plus family and clinic programs). PMID: 27789250 [38]

Although a growing number of publications describe the general benefits of stakeholder engagement in health research [7, 12–14], these tend to discuss the benefits in general terms such as “improve recruitment” or “increase retention,” largely failing to capture the specific—and often illuminating—interactions through which stakeholder input identifies problems, needs and potential solutions, which then lead to specific protocol and implementation changes. We attempt, within the context of engagement literature, to address this gap. The objective of this report is to describe, using numerous examples of stakeholder input, the processes, outcomes, benefits, and challenges of stakeholder engagement in these eight studies of asthma in communities underrepresented in research.

## Methods

### Data sources

The eight comparative effectiveness asthma teams were notified of their awards in late 2013 and funded in early 2014. Table 1 lists the eight research teams, location, target population, study designs and corresponding study names that are referenced throughout this report. Study activities, including the first year of stakeholder engagement, clinical trial operations, and submission of PCORI final reports, were conducted between 2014 and 2021. The research teams were required to involve patients and other stakeholders as research partners in every element of study, including the planning and the conduct of the study. PCORI’s Advisory Panel on Patient Engagement (see here for current and past panel members: https://www.pcori.org/about/pcoris-advisory-panels/advisory-panel-patient-engagement), developed an engagement rubric to provide guidance on engaging stakeholder partners throughout each phase of a research study (Table 2). Using a method of content analysis, we performed a case study to explore each study group’s experiences with stakeholder engagement in their respective studies.

**Table 2:** PCORI engagement rubric, which lists key ways in which patient and stakeholder partners can participate during the three main phases of a study

Planning of the study	Study conduct	Dissemination of study results
Develop the research question and relevant outcomes to be studied to ensure that the project will be stakeholder communities Define the characteristics of study participants to minimize exclusion due to criteria that are not relevant Design the study to minimize disruption to study participants, thereby promoting retention	Draft or revise study materials and protocols to ensure feasibility for clinicians and patient participants Participate in recruitment of study participants to increase and sustain recruitment and ensure viability of the study Participate in data collection and data analysis to lend unique and varied perspectives on interpretation of the data Participate in the evaluation of patient and stakeholder engagement to ensure authenticity and value of engagement Serve as a patient representative on a data safety monitoring board to make the DSMB composition more robust and patient centered	Identify partner organizations for dissemination to ensure meaningful and direct connections with end users Plan dissemination efforts, shaping study design and protocol from the very beginning to be focused on the final product Participate in dissemination efforts, such as authoring manuscripts and presenting study findings, to offer the patient and stakeholder perspective and to reach new and different audiences Identify opportunities to present or share information about the study, even as it is in progress, to move away from traditional models of dissemination and think more creatively about how to get information into the hands of those who need it

Adapted with permission from PCORI: https://www.pcori.org/sites/default/files/Engagement-Rubric.pdf [31]

This study utilizes three primary data sources (1) minutes and discussions from monthly meetings of the eight teams; (2) a survey of the eight research teams regarding their engagement activities; and (3) review of each teams’ formal final research report published by PCORI.

#### Meetings

Early after funding, the eight teams chose to meet monthly to discuss issues, harmonize protocols when possible, and in other ways improve the individual protocols, increase the likelihood for later meta-analyses, and discuss problems and potential solutions. The value of these meetings became apparent, with subsequent PCORI support. These meeting indirectly led to PCORI establishing the Asthma Evidence to Action Network (AE2AN). Engagement and stakeholder suggestions were frequent topics of discussion.

#### Survey

The principal investigators or another representative from the research team completed a structured survey about engagement activities in 2017 and 2019 with input of their research staff members. They were asked to provide as much information as they would like for each domain in the survey and were asked to provide additional information during monthly meetings if the content was not clear. The responses during the calls were captured and entered the survey response. The survey was developed by KS based on the knowledge of the topic, review of the literature and finalized with input of the other PIs based on the consensus of domains that would capture the engagement activities The domains included detailed researcher description/perception of the stakeholder engagement such as type of stakeholder, methods of engagement, research process in which stakeholders were involved, what was changed, and challenges and solutions to stakeholder engagement, whether there was already an established relationship with stakeholders, how stakeholders were identified and recruited, perceived and measured benefit of stakeholder engagement, and if there was an effect on research outside of the PCORI funded project (Additional file 2).

#### Engagement section of final research report

In addition to this survey, we reviewed the final research reports from each study published on PCORI.org, focusing on the required description of engagement activities and participation of patients and other stakeholders [15–22]. The PCORI final research report for each study describes the background, methods, results, and discussion, as well as a summary statement for the general audience. It undergoes extensive editing and quality control that includes a peer review process and is posted on the PCORI site for public access. Final research reports must include a section titled “Participation of patients and other stakeholders in the design and conduct of research and dissemination of findings” [23]. This section systematically describes the engagement activities of a study as directed by the engagement rubric and must include (1) type and number of stakeholders involved (2) how stakeholders were recruited (3) how and why the composition was chosen (4) methods, modes, intensity of engagement activities (5) perceived or measured impact, and (6) specific examples of changes occurred during the study.

### Analysis

We primarily assessed the responses to the surveys and the information provided in the final research reports to categorize the information and to identify similarities and differences across the eight studies. In most instances, data from quarterly reports and meeting minutes were used by the individual teams in responding to the survey and preparing the final report and therefore was redundant for purposes of this study. The specific information extracted and analyzed from each research group’s survey and the PCORI final reports were as follows: (1) types of stakeholders engaged and how they were recruited; (2) specific methods used to engage patients; (3) stage of involvement; (4) details of examples of changes made based on stakeholder feedback; (5) barriers, challenges, and solutions; and (6) perceived benefit of stakeholder engagement.

We analyzed the collected data using simultaneous deductive and inductive content analysis, as described by Elo and Kyngas [24]. Jauch et al. [25] has described the method of using a content analysis schedule to draw relevant information from published case materials in organizational research. Content analysis emphasizes an integrated view of texts, which we felt aligned with our goal to explore meanings, themes, and patterns regarding stakeholder engagement. The data were sorted by deductive categories. We used inductive content analysis to allow for open/unstructured coding to identify emergent or unexpected themes. We also conducted iterative cycles of data collection and organized the data using Excel. After careful review of the surveys and the final reports, along with a review of meeting discussions for any missing data, TD analyzed and coded the content. Initial codes generated were organized into preliminary themes that were developed and refined through discussion with the second author (KS) and reviewed by the third author (BK). Frequencies for quantitative variables were calculated. Findings were also summarized by documented patterns and themes. Data tables were reviewed, interpreted, and further summarized by the authors.

Given the largely qualitative and retrospective nature of our data, the rigor of our data collection and analysis was improved by utilizing (1) multiple researchers to code and review the summary materials, (2) the full research team to review the data collected and subsequent decisions regarding analysis and interpretation, and (3) crosschecks to ensure thorough data collection and organized documentation of each data point.

## Results

We present how stakeholders were being engaged in all phase of our research with specific examples of what was changed based on their feedback, and various barriers to effective stakeholder engagement and proposed solutions identified.

### Description of stakeholders

#### Type of stakeholder

**Fig. 1:**
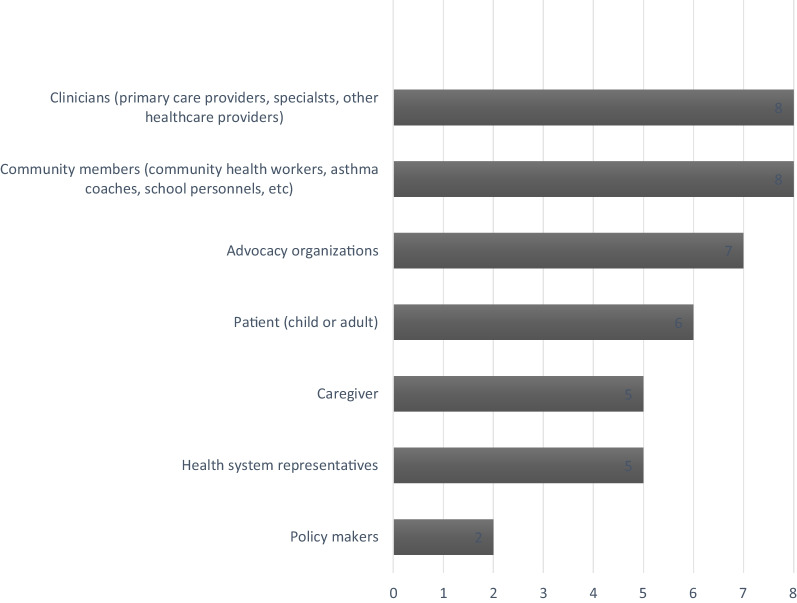
Types of stakeholders identified in the survey by the eight study sites

The most common stakeholders were individuals from target populations (African Americans or Latinos) who were impacted by asthma and thus the study aims: patients with asthma, their caregivers, and healthcare providers (Fig. 1). Local government, policymakers, community advocates, asthma coaches, community health care centers, housing specialists, and the faith community were also represented. Stakeholders also came from community-based organizations (CBOs), and organizations such as the American Lung Association and Respiratory Health Association.

#### Stakeholder recruitment

Most stakeholders were recruited with the goal of representing each study’s target population as well as relevant community leaders and organizations. For example, HIITBAC researchers sought involvement from individuals who would have a vested interest in the study, such as African American adults with asthma who utilized the Harris Health System and community leaders of color. Another important strategy for recruiting stakeholders was utilizing pre-established relationships between researchers and stakeholders. One notable example was the partnership between the Seattle and King County health department and the Allies against asthma coalition, which included representation from schools, public health and housing agencies, academic institutions, community clinics, health providers, residents, and community organizations. In the BEAMS study, researchers identified local community providers based on history of service and advocacy. The ASIST group identified stakeholder participants from an existing community advisory board at their institution, a practice-based research network consortium of community pediatricians. Additionally, two asthma coaches who had children with asthma were included on the ASIST research team. These two asthma coaches had participated in previous asthma research studies and had experience with patient education, which was an essential component of the planned study.

Both the researchers in the SAMBA and CHICAGO studies reported utilizing “snowball sampling,” whereby existing stakeholders assisted in identifying and recruiting additional stakeholders. In New York, the SAMBA team identified representatives from community-based organizations (CBOs) that provide home based asthma support and health coaching through snowball sampling. They also recruited stakeholders from organizations such as the Institute for Family Health (an FQHC that provides guidance on culturally concordant, evidence based care coaching, and also served as centers of recruitment), the Greater New York Hospital Association (GNYHA, who provided guidance on sustainable program dissemination and features that would appeal to health care systems), New York State Office of Quality and Patient Safety (facilitated access to hospitalization and ED visit data, and provided access to more stakeholders), and the New York State Asthma Coalition Network, as well as various individuals from the NY State and City departments of health.

### Methods of engagement

**Fig. 2:**
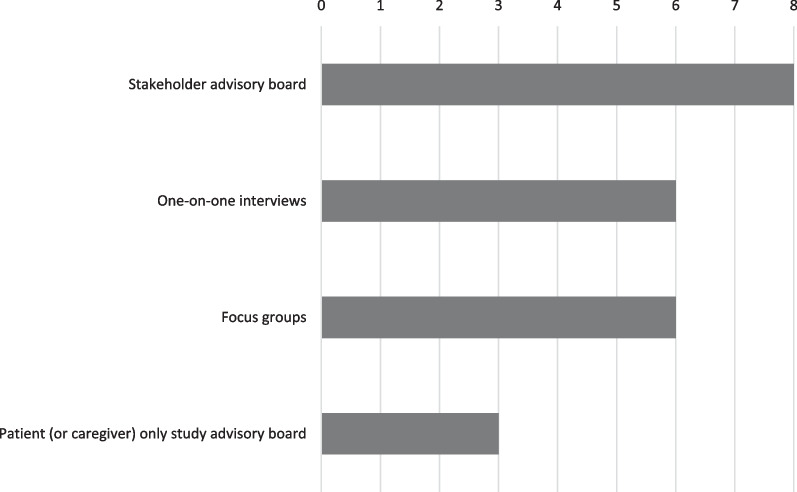
Method of stakeholder engagement, from survey data, among the eight asthma

The most common method of engagement was participation in advisory boards (Fig. 2). Another common method of engagement was the use of focus groups, as demonstrated by the Respira Sano study, in which potential participants from the target study population participated in focus groups prior to and during the planning and preparation of the application. Other reported types of engagement included provider interviews, meetings, one-on-one interviews and “observations in situ.” Depending on the study circumstances, stakeholders were involved as early as possible with some stakeholder groups involved in the planning stages, before or during the application process, whereas others primarily became active immediately after the awards were announced, during the early protocol refinement stages.

#### Stages of involvement

In accordance with the PCORI stakeholder engagement rubric, researchers reported engagement activities in all phases of research: planning, conduct, and dissemination (Table 2). Researchers responded to the survey and in the final research reports described the details of stakeholder engagement activities in all phases of their respective studies.

### Stakeholder-driven changes based on stakeholder feedback by study phase

#### Planning phase

In all eight studies, stakeholders provided input during the planning phase (Table 3). This input led to (1) refining of study questions, outcome measures, and inclusion and exclusion criteria; (2) refining study intervention (design and component of intervention, (3) identifying partner organizations and healthcare providers for studies; and (4) adjusting study-visit structures such as location, frequency, and study visit procedures.

**Table 3:** Study planning: method of engagement and study activities by stakeholder type

Stakeholder	Method of engagement	Study activity	Specific example of what was changed
*Patients with asthma*			
	Advisory group meetings Multi-disciplinary advisory boards	Outcome measure Intervention	Discussions of goals and priorities to develop research question and strategize how community health workers could improve asthma outcomes (G2P study) Developed training and coach certification program (SAMBA)
*Caregivers of children with asthma*			
	Stakeholder/patient “work groups” Asthma coaches Focus groups Focus groups + one-on-one interviews (BEAMS)	Intervention Intervention Outcome measure Intervention, outcome measure	Intervention changed from “usual care” to “enhanced usual care so that all patients benefit from participating in study (CHICAGO) Developed coaching manual that would be used to deliver study intervention (ASIST) Developed intervention (use of CHWs and patient portals) and pilot studies based on information from focus groups [19] De-emphasis on technology and mobile health monitoring as part of intervention for parental stress management and primary outcome measure changed from adherence to symptom free days to reflect priorities of caregivers
*Clinicians*			
	Advisory board (HIITBAC) One on one interviews (ASIST) National advisory core group (BEAMS)	Study design Intervention Study design	Identified primary care provider practice sites to participate in the study Modified inclusion/exclusion criteria to match study population in the community Recommended asthma coaches as part of intervention to provide patient education (ASIST) Investigators who were experts in asthma trials among at risk youth refined study question, outcomes, and intervention
*Community members*			
	CHWs and design strategists in stakeholder/patient “work groups”	Study design, intervention	Developed prototype for in-home asthma education tool that would reflect “real world” settings in which CHWs must adapt (CHICAGO plan)
*Advocacy*			
	Chicago RHA as part of patient/stakeholder workgroup	Study design	Helped identify patient stakeholders who would refine intervention, select outcomes (CHICAGO)

Community health worker (CHW); Repiratory Health Association (RHA)

Stakeholder input during the initial study-design process also resulted in changes that researchers felt increased the feasibility and acceptability of the individual research studies by the target populations and therefore increased the likelihood that potential participants would choose to participate. For example, in the ASIST study, stakeholders made suggestions regarding the appropriate amount of reimbursement for participation in the study and highlighted how providing free study medications would motivate families to participate. As another example, in their study evaluating how well caregivers are prepared to manage their children’s asthma following an emergency department (ED) visit, Krishnan et al. reported that stakeholder input resulted in modifying the study name from the “CHICAGO Trial” to the “CHICAGO Plan,” based on concerns that the word “trial” was associated with civil or criminal court proceedings. Researchers in the BEAMS study planned to evaluate the effect of a parental stress management program that incorporated mobile health monitoring; however, some stakeholders expressed strong reservations to having their medication use monitored electronically or to participating in an intervention that used technology for communication rather than building relationships and personal connections. Therefore, based on this response and feedback from their stakeholders, it was decided to de-emphasize the use of technology in the study.

Stakeholders helped to improve interventional processes as well as the outcome measures studied so that they were more meaningful for the various target populations studies and aligned with the values of patient-centered outcomes research. In the CHICAGO plan, for example, stakeholders felt that a control group receiving “usual care” for asthma was not acceptable because of known gaps between practice (usual care) and current evidence and standard of care evidence. They also noted that recruitment and retention would likely suffer if one in three participants with asthma would not benefit health wise from study participation. The control group was thus redefined as an “enhanced usual care” group, with a baseline of asthma care acceptable to stakeholders. In the BEAMS study, stakeholder input led to a significant change in the original primary outcome measure from adherence to patient symptom free days as this was considered a more meaningful patient-centered outcome. This was based on feedback from parents of children with asthma, whose priorities based on adherence; rather, their goals were for their children to be less symptomatic and less limited by asthma. In the ASIST study, parents of children with asthma participated in pre-study focus groups and confirmed that the proposed intervention of intermittent steroid use aligned with caregivers’ goal to reduce asthma medications. Community provider stakeholders who would be delivering the interventions identified barriers to implementation of some of the proposed interventions noting, for example, there could be confusion among the enrollees between intermittent and daily steroid use; their feedback resulted in the addition of asthma coaches to provide patient education during the trial.

#### Conduct phase

In all eight studies, stakeholders were deeply engaged in recruitment and retention efforts, as well as implementation of the study interventions (Table 4). Six of the studies reported that they adjusted recruitment methods significantly based on stakeholder feedback. Four of the study teams specifically mentioned fine-tuning language and wording of the study questionnaires. One investigator felt that stakeholder input resulted in significantly improved clarity and comprehension of the study and recruitment materials. Stakeholders helped with troubleshooting, such as addressing increasing ongoing enrollee contact between visits (e.g., additional calls, birthday cards, and appointment reminders) to help reduce “no shows,” which were an issue for all the studies. Input from stakeholders also significantly improved recruitment. For example, based on specific suggestions from their Patient/Stakeholder Advisory Council, the HIITBAC study team began recruiting at food distribution sites and reached out to prominent African American churches that were willing to announce the study at Sunday services. In addition, their stakeholders used community contacts to set up interviews about the study with local television and radio stations popular with the targeted population. This research team also described how, based on stakeholder suggestions, the recruitment materials were revised to better target two key reasons why people within Houston’s African American community sign up for clinical trials: (1) individuals particularly interested in improving their asthma and receiving monetary compensation and asthma supplies, and (2) those particularly interested in helping to address the disproportionate burden of asthma in their communities and in African Americans more generally. The CHICAGO study was designed to take place in the ED while children with uncontrolled asthma received care. This was acknowledged as a challenging setting in which to conduct research. Onsite observations by CHICAGO’s stakeholders identified several approaches to engaging children and caregivers to improve recruitment in a busy ED setting. The study was modified to shift recruitment, enrollment, randomization, and intervention activities to the treatment and observation periods during the patient’s ED stay, rather than at discharge when patients and their families are often anxious to leave. The community advisory board in the study by Apter et al. [19] proposed driving to participants’ homes (“drive-ups”) if they were not reachable by phone to improve retention of difficult-to-reach participants.

**Table 4:** STUDY CONDUCT: Method of engagement and study activities by stakeholder type

Stakeholder	Method of engagement	Study activity	Specific examples
*Patients with asthma*			
	Patient/stakeholder advisory board (HIITBAC) Multidisciplinary advisory board Patient-only advisory board	Recruitment and retention Recruitment and retention Recruitment and retention	Recommended strategies to engender trust: use of car magnets, identification/type of clothing of research team Interacted with potential participants at food distribution sites, African American churches, and through interviews with local TV and radio stations Recruitment materials revised to better target African American communities’ motivation for participating in clinical trials Suggested increased frequency of reminders, more compensation options Proposed driving to participant’s home if not reachable by phone [19] Conducted monthly meetings for coaches to improve skills and expand knowledge base (SAMBA) Redesigned recruitment strategies, such as by tailoring recruitment scripts, recommending that tote bags be provided (SAMBA)
*Caregivers of children with asthma*			
	Caregivers as asthma coaches	Delivery of intervention	Asthma coaches designed intervention as well as delivered patient education as part of intervention (ASIST)
*Clinicians*			
	Multi-disciplinary workgroups Community providers as investigators One-on-one interviews	Adjustment of intervention Delivery of intervention Recruitment	On site observations resulted in shifting of study activities (recruitment, enrollment, randomization, intervention) to the treatment and observation period during patient’s ED stay, rather than at discharge (CHICAGO) Recruited patients and administered intervention to their own patients (ASIST) Study manuals, and education and recruitment materials modified to improve recruitment (ASIST)
*Community members*			
	Community health workers as part of “CHW coordinating center”	Direct delivery of intervention	Suggested that community health worker term be changed to “community asthma educator”
*Health care organizations*			
	Institute for Family Health, an FQHC	Recruitment, intervention	Contributed to chronic care model-based health coaching, EHR modifications for asthma clinical decision support, and as recruitment sites

Community health worker (CHW); emergency department (ED), Federally Qualified Health Center (FQHC), electronic health record (EHR)

In some instances, stakeholders were involved in implementing the intervention that they were also directly involved in designing. For example, in the ASIST study, asthma coaches were involved in both planning and conduct of the study. During the planning phase, they provided input on the coaching manual that they would use as a guide to deliver patient education during the study. This study also engaged health care providers from the community (pediatricians and nurse practitioners) as stakeholders who served on advisory boards and subsequently recruited from and administered the study intervention to their own patients.

#### Dissemination phase

In general, dissemination activities reported by the research teams included distribution of results to the wider community and broader stakeholders, preparation of manuscripts, and implementation of the intervention components into the health system (Table 5).

**Table 5:** STUDY DISSEMENINATION: Method of engagement and study activities by stakeholder type

Stakeholder	Method of engagement	Study activity	Specific examples
*Patients with asthma*			
	Patient/Stakeholder Advisory board (HIITBAC)	Patient education	On “World Asthma Day” hosted a town hall to disseminate preliminary study results, conducted a broad discussion of asthma, and delivered list of specific action to City Council on how to make Houston more lung health through interviews with local TV and radio stations (HIITBAC)
*Caregivers of patients with asthma*			
	Parent advisory council (BEAMS)	Future research	Developed parent advisory council to enhance subsequent research and programmatic initiatives BEAMS Shared insights with other research teams considering similar engagement strategies to increase patient centered approaches
*Advocacy organizations*			
	Multi-disciplinary stakeholder committees and meetings with the GNYHA (SAMBA)	Implementation	Created toolkit and implementation guide for organizations adopting and implementing self-management support program Guidance regarding implementation and sustainability of programs Increasing appeal of program implementation to other health care systems
*Health care organizations*			
	Meetings with representatives from NYS and city DOH Representatives from FQHC	Implementation Implementation	Connected research team to health plans, hospitals, academic institutions, professional organizations, community coalitions (SAMBA) Ensured sustainability of intervention by providing ongoing training and support, institutionalizing asthma care plan (asthma education by CHWs), and implementing a quality assurance monitoring system (Respira Sano)
*Community members*			
	Community health workers Multidisciplinary stakeholder group (SAMBA)	Implementation Contextualization of results	Participated in statewide assessment of asthma home visiting programs in partnership with California Department of Public Health (Respira Sano) Redeveloped messaging frames for various local and national audiences Served as community spokespersons to share the study, findings, and broader implications for underrepresented persons with asthma
*Clinicians*			
	National advisory core group (BEAMS)	Contextualization of results	Helped contextualize the study and inform members of the target community about the degree to which study was shaped by patients to hopefully increase acceptance of study findings

Greater New York Hospital Association (GNYHA), New York state (NYS), Department of Health (DOH); Federally Qualified Health Center (FQHC)

Based on input from the HIITBAC study advisory board, dissemination efforts were initiated at the beginning of the study in parallel with recruitment, as opposed to the end of the study, as the stakeholders felt that broad-based education about guidelines-based asthma care and in-home interventions would benefit the health of African Americans more generally, as well as increase enrollment. The BEAMS research team recruited national experts who comprised a national advisory core that helped the study team inform the targeted community and others about the degree to which the study was shaped by patients and others of the targeted community, thereby hopefully increasing the interest in and acceptance of key study findings by this community.

Other researchers reported that stakeholders helped to edit and contextualize the study results in ways that were more understandable for different target audiences. Stakeholders in the SAMBA study, for example, redeveloped messaging frames for various local and national audiences, with some individuals from the stakeholder group serving as community spokespersons to share the study, its findings, and the study’s broader implications for underrepresented persons with asthma. Researchers from the BEAMS study reported that their stakeholders re-framed the study results in a way that was more useful for key decision makers, including parents and providers.

Another component of the dissemination phase is outreach and collaboration. The BEAMS study committed to engaging with parents of children with asthma by developing a Parent Advisory Council to enhance subsequent research and programmatic initiatives. They also shared insights with other research teams considering similar engagement strategies to increase patient centered approaches in clinical research. To ensure that the intervention was sustained following completion of the Respira Sano study, researchers worked with representatives from a Federally Qualified Health Center (FQHC) for migrants to provide ongoing training and support, and to institutionalize an asthma care plan that included asthma education by patient-care coordinators and community health workers (CHWs) and a quality assurance monitoring system. Additionally, the CHWs participated in a statewide assessment of asthma home-visiting programs in partnership with the California Department of Public Health.

### Barriers, challenges, and solutions

The eight research teams described the challenges they encountered with stakeholder engagement and offered solutions for barriers related to (1) stakeholder recruitment, (2) maintaining stakeholder enthusiasm and participation, and (3) forming mutual partnership (Table 6).

**Table 6:** Challenges encountered and solutions utilized to improve stakeholder engagement, by category

Challenges	Solutions
*Recruiting and retaining stakeholders*	
Identifying stakeholder partners Turnover of stakeholder members on the advisory board	Word of mouth Assistance from community leaders or organizations to recruit stakeholders Use of existing infrastructure for community engagement Recommendation by current stakeholders to replace stakeholders who have left
*Maintaining enthusiasm and participation*	
Varying levels of enthusiasm in engagement activities Competing priorities among stakeholder members Deviation of the discussions beyond the scope of the project Lack of participation Difficulty in finding common location Inconsistent attendance due to busy schedule, or patient partner's health	Define expectations upfront Set clear purpose and value for each meeting Have experienced facilitator lead the advisory board meetings To increase youth members’ participation, ask their input first before adult members Provide meals and honoraria at meetings Send meeting minutes, project updates, made study materials accessible to stakeholders via Dropbox Conduct meetings in Spanish Start and end meeting on time Allow remote options for attendance (phone, video conference)
*Maintaining mutual partnership*	
Ensure that stakeholder input is reflected and had true influence in the project Gain mutual benefit from collaboration	Absolute transparency with the stakeholders to show that their work leads to measurable change Invite advisory board member to the research team Provide brief presentations on asthma education relevant to stakeholders and their families

Stakeholder recruitment was one of the most common challenges that researchers encountered. The CHICAGO research team felt that utilizing pre-existing relationships with the Respiratory Health Association and the Chicago Asthma Consortium helped to streamline stakeholder engagement and quickly engage and sustain input from relevant community stakeholders. Teams that used a study-specific stakeholder group tended to use clinicians providing asthma care to the targeted community to help identify patients who did not qualify for the study but might be interested in several on an advisory council; some teams also brought on board patients once they completed enrollment in the study.

A common challenge noted by three study groups was the varying degrees of enthusiasm or competing interests of stakeholders. For example, some advisory meeting agendas were more compelling for youth members, such as brainstorming and reviewing materials, whereas other agenda items, such as monitoring protocols, were less compelling. To engage youth members, the Respira Sano team addressed questions and elicited feedback from them first, before adult members, during advisory board meetings. ASIST researchers gave brief educational presentations during advisory board meetings about asthma topics that they believed stakeholders and their families would find relevant and informative. Another challenge noted by the Teach group was the difficulty of responding satisfactorily to suggestions that could not be implemented, perhaps because the suggestions were beyond the scope of the project or because of budgetary constraints. Researchers suggested defining a clear purpose and expectations for each meeting. The HIITBAC used a paid meeting facilitator not associated with the research team to conduct the meetings. This person oversaw the agenda, used stakeholder principles to establish rules of respect and techniques (e.g., no professional titles) to ensure that each stakeholder’s input was expressed and heard, and made certain that meetings stayed on task and started and ended on time. To address the language barrier, Respira Sano study stakeholders participated in quarterly meetings conducted primarily in Spanish, with meeting minutes available in English and Spanish. Four studies (50%) reported that finding common meeting locations and times was a significant challenge. Two researchers mentioned that allowing stakeholders to phone or video into the meetings from a remote location was helpful for improving attendance. Other solutions included evening meetings, providing transportation, reimbursement for gas, stipend and meals and babysitting was provided if needed.

Another challenge was maintaining mutual partnership between researchers and stakeholders. HIITBAC researchers helped advisory board members with events and funding opportunities, and HIITBAC stakeholders were encouraged to become active in national PCORI engagement activities and report on them at meeting as well as represent the study at community events and on local media. Most of the studies included the stakeholders as coauthors of one or more or the resultant journal publications.

### Perceived benefit of stakeholder engagement

Each of the eight teams felt that the degree of stakeholder engagement and openness to rethinking the initial protocols and implementation processes led to significant measurable changes in their study that improved the study and highlights the value of a meaningful and transparent partnership with stakeholders. In addition, the teams felt that populations underrepresented in clinical research are often largely underrepresented because a trusting relationship between the target community and the medical establishment largely does not exist. For these communities, partnering with stakeholders from these communities or who are trusted within the communities can be critical in building relationships that not only help with immediate study enrollment but help to grow connections that can facilitate future research.

## Discussion

In our analysis of the eight studies funded by PCORI’s asthma disparities program, we identified numerous stakeholder-driven enhancements in the planning and conduct of each study. Although the studies varied in population demographics, geographic locations, and specific study questions, we identified common themes with all research teams reporting that stakeholders improved study designs, study materials, recruitment strategies, and dissemination methods. The most common stakeholder types were clinicians, caregivers and individuals with asthma who represented the target demographics of the study. Community members and advocacy groups were also represented. The most common modes of stakeholder engagement were advisory board memberships or participation in interviews or focus groups. Stakeholder insights resulted in revised study protocols that increased acceptability among potential study participants and in the broader community.

Addressing barriers early on can facilitate stakeholder engagement [26]. Researchers who engage stakeholders in asthma research might benefit from anticipating challenges similar to those we identified in this report. One obstacle that all eight teams encountered was recruitment of stakeholders. Similar to the challenges inherent in recruitment for research in economically disadvantaged and underrepresented populations, these same factors may contribute to barriers observed with consistent stakeholder participation, such as language barriers and lack of transportation. Examples of barriers to participation of minority groups in research include mistrust, fear, lack of confidence, and logistical concerns such as childcare, schedule conflicts, lack of transportation, and lack of adequate information about clinical research [27]. In this study, we share specific examples in which stakeholders provided real world perspectives and, ultimately, strategies to improve recruitment and retention of study participants.

Another challenge that we encountered was competing interests or goals and varying degrees of enthusiasm. This is consistent with previous reports of stakeholder engagement experience in comparative effectiveness research. Han et al., for example, noted that priorities, motivations, and ways of working in focus groups often differ between researchers and community partners, leading to conflict and power struggles [28]. Strategies to maintain enthusiasm and promote mutual partnership were suggested by all eight asthma study teams, including establishing a clear agenda and expectations for each stakeholder meeting, and clearly communicating with stakeholders how their involvement leads to measurable change. Based on our collective experience, focus groups are valuable for gathering stakeholder perspectives. However, we suggest that researchers anticipate the potential for uneven power dynamics that might develop in focus groups. We suggest considering additional methods of working with stakeholders in groups that eliminate this power dynamic to engage them as mutual partners, for example as members of advisory boards or directly as research team members.

In developing this report, we reflected on the unique opportunity to exchange ideas for recruiting and involving stakeholders in research studies of underrepresented and underserved populations that are disproportionately affected by asthma. A commitment to stakeholder engagement was required by PCORI’s funding initiative for these asthma studies, requiring that researchers integrate patients and stakeholders meaningfully in all phases of the proposed project. This included formulation of research questions, defining essential characteristics of the study, monitoring study conduct and progress, and disseminating research results. PCORI’s initiative to fund eight studies of asthma in African Americans and Latinos allowed the eight research teams with similar objectives to collaborate and identify barriers and challenges to stakeholder engagement early on. Therefore, we feel that stakeholder engagement in these studies was unusually robust and unprecedented relative to other comparative effectiveness research that is typically initiated and driven by researchers. Although at times challenging, at the end of each project the teams all felt that stakeholder engagement was an exceptionally valuable and yet under-utilized approach for planning and executing research studies in which asthma patients from these underrepresented communities would be motivated to participate and contribute.

There are some limitations to our analysis. One limitation is that the data are largely descriptive and we were unable to assess the impact of the engagement objectively. Staley et al. have advocated for the value of experiential knowledge of patients, and how this makes clinical data relevant [29]. In contrast, Goodman et al. advocate for rigorously evaluating the impact of stakeholder engagement on the development, implementation, and outcomes of research. In their study, a five-round modified Delphi process was utilized to arrive at a core set of engagement principles that could be used as a quantitative measure of meaningful stakeholder engagement [30]. This quantitative measurement was not available at the time of our study to quantify the impact of the engagement, but we used the PCORI engagement rubric to systemically report stakeholder engagement activities during all phases of the studies [31]. Our report summarizes how the eight research teams applied these principles, and the subsequent impacts of this engagement process.

Another limitation of this report is that the perceived impact was primarily based on the researchers’ point of view. Although we have not directly included stakeholders’ point of view in this report, researchers were required to submit research updates, which included a section on stakeholder engagement, throughout the duration of their respected studies. Stakeholder perspectives were incorporated in these research updates, primarily through review of stakeholder meeting agendas and direct quotes that were recorded. A summary of stakeholder perspectives was also included in the final research reports that were ultimately used to compile the observations reported here. Future analyses might directly query stakeholder reflections regarding the impact of their engagement after the completion of a research study.

A growing body of literature details how stakeholder engagement has resulted in valuable contributions to research feasibility, acceptability, rigor, and relevance in research for conditions such as chronic pain, cancer treatment, and infectious diseases [32]. Our report indicates that this is also true of the impact of stakeholder engagement on studies of asthma, specifically in populations that are underrepresented in research. As noted by one study team, “Patient contributions to the design and implementation of the study were especially valuable, and development of these relationships convinced us of the need to engage them in all future research activities.” Knowledge sharing and learning between research teams was also considered beneficial. The strengthened relationships between research teams and the community may help overcome challenges that have long been barriers to research for the very individuals and communities that this research targets.

## Conclusions

Stakeholder engagement activities led to study designs that had increased support and acceptance in communities underrepresented in asthma research. Compared with traditional health research, outcomes were considered more meaningful and applicable to these populations and were more aligned with goals of patient-centered outcomes research. This level of investigator-stakeholder interaction is unprecedented, but there is an increasing demand to engage stakeholders systematically in patient-centered outcomes research. Despite the barriers and challenges that were encountered, stakeholder engagement was considered beneficial and enhanced the rigor of the asthma studies included in this report.

## Supplementary Information

**Additional file 1**. AAMC Principles of Trustworthiness (9).

**Additional file 2**. Survey questions for stakeholder engagement.

## Data Availability

All data generated or analyzed during this study are included in this published article. That datasets used and analyzed during the current study are available from the corresponding author on reasonable request.

## Abbreviations

AAMC: Association of American Medical Colleges
AE2AN: Asthma Evidence to Action Network
ASIST: Asthma Symptom-based adjustment of Inhaled Steroid Therapy in African American children
BEAMS: Breath with Ease: A Unique Approach to Managing Stress
CBO: Community based organizations
CHW: Community health worker
CHICAGO: Coordinated Healthcare Interventions for Childhood Asthma Gaps in Outcomes
ED: Emergency department
FQHC: Federally Qualified Health Center
G2P: Guidelines to Practice: Reducing Asthma Health Disparities through Guideline Implementation
HIITBAC: Houston home-based integrated intervention targeting better asthma control for African Americans
PCORI: Patient-Centered Outcomes Research Institute
Respira Sano: Comparing Programs to Improve Asthma Control and Quality of Life for Latino Youth Living in Rural Areas and Their Caregivers
SAMBA: Supporting asthma self-management behaviors in aging adults
