# Supplementary material for: Stakeholder engagement in eight comparative effectiveness trials in African Americans and Latinos with asthma
Source: Res Involv Engagem. 2022 Nov 24;8:63. doi: 10.1186/s40900-022-00399-x (PMC9694541; doi:10.1186/s40900-022-00399-x)
Supplement: Supplementary file 1 — Additional file 1. AAMC Principles of Trustworthiness (9). [file 40900_2022_399_MOESM1_ESM.docx]

**Additional File 1.** AAMC Principles of Trustworthiness (9)

| **Principle** | **Description** |
| --- | --- |
| **The community is already educated; that’s why it doesn’t trust you.** | Words matter. Be mindful of how you frame your relationship. It is not your job to teach to the gaps you assume the community has. Mistrust is a rational response to actual injustice. The community knows what it doesn’t know and will ask when it thinks you have answers it can trust. |
| **You are not the only experts.** | People closest to injustice are also those closest to the solutions to that injustice. (That is probably not you or your organization and, even if it is, there’s a power imbalance.) Listen to people in your community. They have deployed survival tactics and strategies for decades – centuries, even. Take notes. Co-develop. Co-lead. Share power. |
| **Without action, your organizational pledge is only performance.** | Walk the walk, please. Deploy resources. Coordinate across your organization. Hire someone to the C-suite and a network or coalition of experts to be responsible for transformation because transformation is not a one-person job. Be authentic. Don’t just say you’re committed to the goal of health equity; do the work to achieve it. |
| **An office of community engagement is insufficient.** | One full-time employee doesn’t cut it. Don’t jam this work into your existing diversity and inclusion office, either. Trustworthiness is not a “minority tax”; we are all responsible. This is systemwide, all-hands-on-deck work and, as such, such be acknowledged, incentivized, and promoted in material ways. |
| **It doesn’t start or end with a community advisory board.** | Running your thoughts by a group of self-appointed community leaders for a thumbs-up does not suffice. Take to the streets to get some unfiltered opinions. And then work together with the community to put that wisdom into the work. Make it clear to all you’ve done so, and explain the benefits accrued. |
| **Diversity is more than skin deep.** | We are diverse within our diversity. Do not rely solely on matching skin tones to make a difference. Think intersectionality and multiple identities but remember: humility and honest are the foundation for earning trust. |
| **There’s more than one gay bar, one “Black church,” and one bodega in your community.** | Not all gay people go to the club, and not all people of color go to the same church (or go at all). Know all of your community’s asses. Visit them. Meet the patrons. Meet the leaders. Break bread and share a meal–at their tables. |
| **Show your work.** | The community does not think you are perfect, and the past is always present. So be transparent about your limitations, your biases, your goals, your funding, and the outcomes that matter to you. Then ask the community to do the same. Identify the “win-win” for all parties. No secrets, no surprises. |
| **If you’re gonna do it, take your time, do it right.** | Demonstrating trustworthiness is not a one-and-done proposition. Keep at it. Be mindful. Remember, it takes a long time to build trust and only a split second to destroy it. Pace yourself. |
| **The project may be over, but the work is not.** | Do not drop in and drop out. Share results. Partner on next steps. Close the loop. The community is constant – it is not there only for the duration of your grant or initiative. Be there for the community, always, and it is more likely to want to be there for you. |
