# Supplementary material for: Stakeholder engagement in eight comparative effectiveness trials in African Americans and Latinos with asthma
Source: Res Involv Engagem. 2022 Nov 24;8:63. doi: 10.1186/s40900-022-00399-x (PMC9694541; doi:10.1186/s40900-022-00399-x)
Supplement: Supplementary file 2 — Additional file 2. Survey questions for stakeholder engagement. [file 40900_2022_399_MOESM2_ESM.docx]

**Additional File 2:** Survey questions for stakeholder engagement.

1. What is the name of the project?
2. Did you have an established relationship with your stakeholders before the grant application?
3. What is the community from which you identified stakeholders?
4. Who were the stakeholders engaged (e.g. patients, caregivers)?
5. Did the stakeholder receive a stipend?
6. What were the methods used to engage patient partners?
7. What were the methods used to engage non-patient stakeholders?
8. In which research process did you engage them?
9. What were examples of the research process or other items that were changed in your study based on stakeholder engagement?
10. What was the composition of stakeholder group meetings? How many meetings occurred and at what frequency?
11. What were barriers encountered? What solutions were placed?
12. What is the perceived or measured benefit of stakeholder engagement?
13. Did the experience in stakeholder engagement through your PCORI project have an effect on research outside of the PCORI project? If yes, what was the unexpected influence?
